# Selection of a Nuclease-Resistant RNA Aptamer Targeting CD19

**DOI:** 10.3390/cancers13205220

**Published:** 2021-10-18

**Authors:** Carla L. Esposito, Katrien Van Roosbroeck, Gianluca Santamaria, Deborah Rotoli, Annamaria Sandomenico, William G. Wierda, Alessandra Ferrajoli, Menotti Ruvo, George A. Calin, Vittorio de Franciscis, Silvia Catuogno

**Affiliations:** 1 IEOS—Istituto per l’endocrinologia e l’oncologia “Gaetano Salvatore”, CNR, 80131 Naples, Italy; c.esposito@ieos.cnr.it (C.L.E.); d.rotoli@ieos.cnr.it (D.R.); vittorio.defranciscis@irgb.cnr.it (V.d.F.); 2 Department of Experimental Therapeutics, The University of Texas MD Anderson Cancer Center, Houston, TX 77030, USA; kvan1@mdanderson.org; 3 Klinikum Rechts der Isar, Department of Regenerative Medicine in Cardiovascular Disease, Technical University of Munich, 81675 Munich, Germany; santamariagianluca@tum.de; 4 Istituto di Biostrutture e Bioimmagini, CNR, 80134 Naples, Italy; annamaria.sandomenico@cnr.it (A.S.); menotti.ruvo@unina.it (M.R.); 5 Department of Leukemia, The University of Texas MD Anderson Cancer Center, Houston, TX 77030, USA; wwierda@mdanderson.org (W.G.W.); aferrajo@mdanderson.org (A.F.); gcalin@mdanderson.org (G.A.C.); 6 Department of Translational Molecular Pathology, The University of Texas MD Anderson Cancer Center, Houston, TX 77030, USA; 7 The RNA Interference and Non-Coding RNA Center, MD Anderson Cancer Center, Houston, TX 77030, USA; 8 IRGB—Institute of Genetic & Biomedical Research—CNR, 20138 Milan, Italy

**Keywords:** CD19, aptamer, B-cells, cell-SELEX

## Abstract

**Simple Summary:**

Haematological malignancies show a constantly growing incidence, accounting for 6.5% of new cancer cases worldwide. Among them, B-cell neoplasms often show resistance to conventional chemotherapy that is also associated with numerous adverse effects. Therefore, in order for the treatment outcome to be improved, the development of new safe and effective targeted therapeutic approaches represents a main challenge. In this regard, nucleic acid aptamers are very attractive molecules. Indeed, they show high affinity and specificity for their target, increased tumour penetration, and low toxicity. Recently, CD19 has emerged as a key surface marker of malignant B cells, suitable for the development of new compounds for malignant B-cell targeting. Here, we isolated an RNA aptamer targeting the human CD19 antigen on malignant B cells that was able to rapidly internalise into target cells. Therefore, it represents a useful carrier for secondary reagents and a promising tool for the development of new safe and effective targeted therapies for B-cell malignancy treatment.

**Abstract:**

The transmembrane glycoprotein cluster of differentiation 19 (CD19) is a B cell–specific surface marker, expressed on the majority of neoplastic B cells, and has recently emerged as a very attractive biomarker and therapeutic target for B-cell malignancies. The development of safe and effective ligands for CD19 has become an important need for the development of targeted conventional and immunotherapies. In this regard, aptamers represent a very interesting class of molecules. Additionally referred to as ‘chemical antibodies’, they show many advantages as therapeutics, including low toxicity and immunogenicity. Here, we isolated a nuclease-resistant RNA aptamer binding to the human CD19 glycoprotein. In order to develop an aptamer also useful as a carrier for secondary reagents, we adopted a cell-based SELEX (Systematic Evolution of Ligands by EXponential Enrichment) protocol adapted to isolate aptamers able to internalise upon binding to their cell surface target. We describe a 2′-fluoro pyrimidine modified aptamer, named B85.T2, which specifically binds to CD19 and shows an exquisite stability in human serum. The aptamer showed an estimated dissociation constant (K_D_) of 49.9 ± 13 nM on purified human recombinant CD19 (rhCD19) glycoprotein, a good binding activity on human B-cell chronic lymphocytic leukaemia cells expressing CD19, and also an effective and rapid cell internalisation, thus representing a promising molecule for CD19 targeting, as well as for the development of new B-cell malignancy-targeted therapies.

## 1. Introduction

CD19 is a B cell-specific type I transmembrane glycoprotein, belonging to the immunoglobulin (Ig) superfamily, which functions as a major co-stimulatory molecule for the amplification of the B-cell receptor (BCR)-dependent responses. CD19 is expressed exclusively on B cells, from the stage of pro-B cells to that of early plasma cells, while it is not expressed on haematopoietic stem cells or on B cells before the pro-B cell differentiation stage [1,2,3]. Importantly, the expression of CD19 is maintained during malignant transformation of B cells, and it is found highly expressed in the majority of B cell-derived malignancies [4,5,6], but not in other normal body tissues or cells. Given the restricted lineage expression, CD19 has emerged as an attractive targetable marker for B-cell malignancy diagnosis and treatment [7,8]. CD19 is, for instance, the target antigen of blinatumomab (Blincyto), a CD19-CD3 bispecific antibody, and of Tisagenlecleucel (CTL019), a CAR-T product, both approved by the FDA for the treatment of relapsed and refractory B cell acute lymphocytic leukaemia (B-ALL) by immunotherapy [9,10,11]. Moreover, given the capacity to induce ligand internalisation, CD19 is also a suitable target for the development of innovative molecules for the targeted delivery of secondary reagents for diagnostic or therapeutic purposes.

Small structured nucleic acid aptamers have emerged as effective tools for targeting relevant markers in cancer pathogenesis, with a great potential for both diagnostic and therapeutic uses [12]. Indeed, aptamers against receptors overexpressed in cancer may specifically recognise their proper target and are generally endowed with an inhibitory activity, competing with the endogenous ligand for the binding to the target receptor. Moreover, aptamers possess many important advantages over monoclonal antibodies, including low immunogenicity, small size, high batch fidelity, easy production, increased chemical stability, and high versatility [13]. All these features render aptamers suitable to receive various chemical modifications for their development and improvement as drugs or molecular imaging probes as well. In addition, in some cases, aptamers against cell surface receptors, upon binding to their proper target, demonstrate to undergo target receptor-mediated intracellular uptake, thus representing effective carrier molecules for the targeted delivery of secondary reagents of both diagnostic and therapeutic interest [14,15].

In this work, we address the generation and the characterisation of a nuclease resistant 2′fluoro-pyrimidine (2′F-Py) RNA aptamer, selectively binding to the human CD19 glycoprotein, as a potential tool for experimental studies, diagnosis, and treatment of B-cell malignancies.

## 2. Materials and Methods

### 2.1. Cell Cultures and Transfection

Used cell lines were from ATCC (LG Standards, Milan, Italy). COS-7, T98G, and U87MG cells were grown in Dulbecco’s modified Eagle’s medium (DMEM), while MEC-1, Jurkat, A549, CLL178, and CLL185 cells were grown in Roswell Park Memorial Institute (RPMI) 1640 medium, both supplemented with 10% of foetal bovine serum (FBS). We added 100 U/mL penicillin/streptomycin for continuous non-primary cell lines. All the transfections were performed by using Lipofectamine 2000 reagent (Invitrogen, Carlsbad, CA, USA), according to the manufacturer’s instructions. The CD19-expressing plasmid vector (CAT#: SC328905) was purchased from OriGene Technologies (9620 Medical Center Drive, Suite 200, Rockville, MD 20850, USA).

### 2.2. Cell-SELEX

The starting aptamer pool (G0) (TriLink BioTechnologies, San Diego, CA, USA) contains 2′F-Py RNAs showing a central degenerated region (45 mer) and two fixed regions at the extremities necessary for the RT-PCR amplification and the in vitro transcription procedure. The forward selection primer sequence was 5′-TAATACGACTCACTATAGGGAGACAAGAATAAACGCTCAA-3′; the reverse selection primer sequence was 5′- GCCTGTTGTGAGCCTCCTGTCGAA-3′. Parental COS-7 cells (CD19^−^) were used in the counter-selection step, while COS-7 transiently transfected with the human CD19 glycoprotein were used in the positive selection step (COS/CD19^+^). The aptamer library was first incubated with COS-7 cells, and then unbound sequences were incubated with COS/CD19^+^ cells. Bound aptamers were recovered, and RT-PCR and in vitro transcription were performed. After eight rounds of conventional cell-SELEX, two additional rounds of ‘cell-internalising SELEX’ were performed. In these two rounds, the library was incubated only with COS-7/CD19^+^, without any prior incubation with non-target cells (counter-selection step), and following five washes with DMEM serum-free medium to remove unbound aptamers, cells were treated with proteinase K (Roche Diagnostics, IN, USA), for 30 min at 0.5 µg/µL final concentration, in order to deplete for aptamers bound to CD19 expressed on the cell surface. Cells were then washed with serum-free DMEM, and internalised RNA aptamers were recovered by RNA extraction. HTS was performed at rounds III, IV, V, VI, VII, VIII, IX, and X with MySeq Illumina sequencer, following the Illumina MySeq sequence preparation. Raw count reads were generated using python in-house script, then imported in R environment to produce the correlation plot [16] (The R Project for Statistical Computing. URL https://www.R-project.org/, (accessed on 21 May 2020).

### 2.3. Aptamer Sequences

B88:
5′-GGGAGACAAGAAUAAACGCUCAAUGAUAGACAUUCGGUGCUCUCUUUCAUUGACCGUUCACCUGUUGUUCGACAGGAGGCUCACAACAGGC-3′
B85:
5′-GGGAGACAAGAAUAAACGCUCAACGUUGACAACAAAUGACGAUCGUCAACAUGAUGCUUGAGCCCUGUUCGACAGGAGGCUCACAACAGGC-3′
B97:
5′-GGGAGACAAGAAUAAACGCUCAACGUGCAACGCACAAAUUCUUGAUCAUCUCAAUGAUGUGUGCUUUCGACAGGAGGCUCACAACAGGC-3′
B146:
5′-GGGAGACAAGAAUAAACGCUCAACGACACGUUGCCAGCCGGAGCCUUAGUAACGUGCUUUGAUGUCGAUUCGACAGGAGGCUCACAACAGGC-3′
B88.T1:
5′-AACGCUCAAUGAUAGACAUUCGGUGCUCUCUUUCAUUGACCGUU-3′
B88.T2:
5′-CCUGUUGUUCGACAGGAGGCUCACAACAGG-3′
B85.T1:
5′-GCUCAACGUUGACAACAAAUGACGAUCGUCAACAUUGAUGC-3′
B85.T2:
5′-UGAGCCCUGUUCGACAGGAGGCUCA-3′
B85.T2 (FAM):
5′-(FAM)UGAGCCCUGUUCGACAGGAGGCUCA-3′
B85.T2 (Biotin):
5′-UGAGCCCUGUUCGACAGGAGGCUCA(BioBB)-3′
Ctrl Apt:
5′-UUCGUACCGGGUAGGUUGGCUUGCACAUAGAACGUGUCA-3′
Ctrl Apt (Biotin):
5′-UUCGUACCGGGUAGGUUGGCUUGCACAUAGAACGUGUCA(BioBB)-3′
scraApt:
5′-GCUCAACGUUGACAACAAAUGACGA-3′
B85.T2 stick:
5′-UGAGCCCUGUUCGACAGGAGGCUCAXXXXGUACAUUCUAGAUAGCC-3′
SCRA 6 stick (SCRA6):
5′-CUUGUCAGUCAAGGAGGGUGCCACCXXXXGUACAUUCUAGAUAGCC-3′
Scra stick (SCRA):
5′-GCUCAACGUUGACAACAAAUGACGAXXXXGUACAUUCUAGAUAGCC-3′
A9g stick (A9g):
5′-GGGACCGAAAAAGACCUGACUUCUAUACUAAGUCUACGUUCCCXXXXGUACAUUCUAGAUAGCC-3′
miR-16 passenger stick:
5′-ACUCCAGUAUUAACUGUGCUGCUGAGGGGCUAUCUAGAAUGUAC-3′
miR-16 guide:
5′-CCUUAGCAGCACGUAAAUAUUGGCGU-3′
miR-15a passenger stick:
5′-CGCAGGCCAUAUUGUGCUGCCUCAUGGCUAUCUAGAAUGUAC-3′
miR-15a guide:
5′-AAGUAGCAGCACAUAAUGGUUUGUGGG-3′
Scrambled miR passenger stick:
5′-UUAUCGUACUAUCACCUAAGAUGCCGGCUAUCUAGAAUGUAC-3′
Scrambled miR guide:
5′-GGCAUCUUAGGUGAUAGUACGAUAAGG-3′

RNA sequences contain 2′F-Py. Aptamers B88, B85, B97, and B85.T2 (Biotin) were synthesised by ChemGenes Corporation (Wilmington, MA, USA) or produced by in vitro transcription. The other aptamer sequences were produced by TriLink Bio Technologies (San Diego, CA, USA) or Synthetic and Biopolymer Chemistry Core at the Beckman Research Institute of City of Hope (Duarte, CA, USA). The stick portions (underlined) contain 2′F-Py and 2′-oxygen-methyl purines. The X indicate a three-carbon linker spacer ([CH2]3).

Before use, aptamers are subjected to the following temperature cycle to ensure their correct folding: 5 min at 85 °C, 5 min on ice, 10 min at 37 °C.

### 2.4. Binding Analysis

Binding assays by qRT-PCR: At 48 h post-transfection, COS-7/Cd19^+^ and parental COS-7 cells were treated with 200 nM aptamer for 30 min at 37 °C in the presence of 0.1 µg/µL polyinosinic acid (Poly(I)) in serum-free medium. Following the incubation, three washings with ice-cold PBS were performed to remove unbound aptamers. Bound RNA aptamers were then recovered with TRIzol in the presence of 0.5 pmol/mL aptamer CL4 (CL4: 5′-GCCUUAGUAACGUGCUUUGAUGUCGAUUCGACAGGAGGC-3′), used as a control for normalisation. The quantity of aptamer bound to the cells was determined by a two-step qRT-PCR procedure. In a first step, the RNA was reverse-transcribed in vitro with specific 3′ primers, according to the following protocol: 5 min at 65 °C, annealing for 5 min at 22 °C, extension for 30 min at 42 °C, end extension for 30 min at 48 °C, enzyme inactivation for 5 min at 95 °C. In a second step, the samples were amplified by real-time quantitative (q)PCR using iQ SYBR Green Supermix (Bio-Rad, Hercules, CA, USA) with the following protocol: 2 min at 95 °C, 40 cycles of heating at 95 °C for 30 s, annealing at 55 °C for 30 s, and extension at 60 °C for 30 s. Further, a melting curve step was performed by heating at 60–95 °C. The sequences of primers used for PCR amplification were Fw 5′-TAATACGACTCACTATAGGGAGACAAGAATAAACGCTCAA-3′ and Rv 5′-GCCTGTTGTGAGCCTCCTGTCGAA-3′ used for G0 starting library and for B88, B85, and B97 long aptamers; primer CL4 Fw 5′-GCCTTAGTAACGTGCTTT-3′ and primer CL4 Rv 5′-GCCTCCTGTCGAATCG-3′; B88.T1 Fw 5′-AACGCTCAATGATAGACAT-3′ and B88.T1 Rv 5′- AACGGTCAATGAAAGAGA-3′; B88.T2 Fw 5′-CGATCCCTGTTGTTCGA-3′ and B88.T2 Rv 5′-AGGCAATACGACCTGTT-3′; B85.T1 Fw 5′-GCTCAACGTTGACAACAA-3′ and B85.T1 Rv 5′- GCATCAATGTTGACGATC-3′; B85.T2 Fw 5′-ACGATCTGAGCCCTGTT-3′ and B85.T2 Rv 5′-AGGCAATACGATGAGC-3′; Scra Fw 5′-TAATACGACTCACTATAGGGAGACAAGAATAAACGCTCAA-3′ and Scra Rv 5′-GCCTGTTGTGAGCCT CCTGTCGAA-3′. Data were normalised to the CL4 reference control and to the number of cells, as determined by counting cells cultured in conjunction with each experiment.

Binding assay by FACS: A total of 3 × 10^5^ cells were washed three times with serum-free medium, resuspended in 300 µL of serum free medium containing 0.4 µg/µL tRNA and 100 nM of Ctrl Apt (Biotin) as unspecific competitors, and incubated for 30 min at 37 °C with slow shaking. Following pre-treatment with the competitors, cells were treated with 250 nM of FAM-labeled B85.T2 aptamer for 30 min at 37 °C with slow shaking in the presence of the competitors, washed once with ice-cold PBS, and then the mean fluorescence was measured at FACS, the cell auto-florescence was subtracted, and the specific binding on CD19^+^ cells was calculated.

### 2.5. Aptamer In Vitro Serum Stability

B88.T2 and B85.T2 aptamers were incubated at 4 µM final concentration in 80% human serum for different times. Type AB Human Serum provided by Euroclone (category ECS0219D) was used. At each time point, 16 pmol of aptamer was recovered and incubated with proteinase K (20 mg/mL) for 1 h at 37 °C to remove serum proteins impairing the electrophoretic migration. Then, the samples were resuspended in a denaturing RNA dye (Invitrogen, Waltham, MA, USA) and loaded on 15% denaturing polyacrylamide gel. Gels were stained with ethidium bromide and displayed under UVs.

### 2.6. Dose-Response Binding by FACS

A total of 3 × 10^5^ MEC-1 cells were washed two times with 5 mL of RPMI serum-free medium, resuspended in 300 µL of serum-free medium containing 0.4 µg/µL tRNA and 100 nM of biotinylated Ctrl Apt as unspecific competitors, and incubated for 30 min at 37 °C with slow shaking. Following the pre-treatment with the unspecific competitors, cells were treated with FAM-labelled B85.T2 aptamer at increasing concentrations (25 nM, 50 nM, 100 nM, 200 nM, 300 nM, 400 nM, 500 nM, 600 nM) for 30 min at 37 °C with slow shaking. Then, cells were washed once with ice-cold PBS, and the mean fluorescence was measured at FACS; cell auto-florescence was subtracted.

### 2.7. Binding and K_D_ Determination by Bio-Layer Interferometry (BLI)

BLI measurements were performed according to the manufacturer’s instructions using a BLItz system and related commercial biosensors (ForteBio, Fremont, CA, USA). Assays were performed through immobilising the human recombinant CD19 (rhCD19) glycoprotein (Sino Biological, Wayne, PA, USA) or the human recombinant B Cell Maturation Antigen (rhBCMA) protein (Sino Biological, Wayne, PA, USA) used as a negative control on the surface of ARG2 sensor chips following the amino coupling procedure. After pre-hydration for 10 min in PBS buffer, the AR2G tips were activated with an EDC (0.2 M)/NHS (0.05 M) coupling mixture for 300 s; then, rhCD19 and rhBCMA were exposed for 180 s to distinct biosensors at the concentration of 10 µg/mL in 10 mM Na acetate at pH 5 and pH 4.5, respectively. Unused activated carboxylated groups on the tip’s surface were reacted with 1.0 M ethanolamine hydrochloride, pH 8.5, for 120 s. After activation, a regeneration step with 10 mM NaOH was performed to minimise non-specific binding. Dose–response binding experiments to rhCD19 were performed with both B85.T2 and with an unrelated control aptamer (Ctrl Apt). Experiments with B85.T2 were performed in the concentration range between 2.5 and 100 nM, while Ctrl Apt was used at concentrations between 10 and 1 µM. As a further control experiment, the binding of B85.T2 to the unrelated protein rhBCMA was performed using the aptamer at concentrations between 10 and 1 µM. Each individual assay was completed through performing the following steps: (i) exposure to running buffer to acquire the initial baseline (baseline, exposure time 30 s); (ii) exposure to protein solutions (association, volume 4.0 µL, exposure time 180 s); (iii) exposure to running buffer (dissociation, exposure time 120 s); (iv) exposure to 5 mM NaOH (three times; regeneration, exposure time 20 s). A reduced volume sample cuvette (4 µL) was used for all the experiments with the shaker speed set to 2000 rpm according to the manufacturer’s instructions. Reference interferograms were subtracted from experimental values before data processing to reduce the background. Data were exported from the BLItz Pro 1.2 software (ForteBio, Fremont, CA, USA) and re-plotted with GraphPad Prism, vers. 5.00, GraphPad Software (San Diego, CA, USA). Plateau values of binding as reflected by changes in optical thickness (nm) at 202 s were used to calculate the affinity constant (K_D_) by applying a non-linear curve fitting algorithm and the one binding site hyperbola as binding model (GraphPad Prism). Data fitting was carried out using the ‘one site binding hyperbola’ algorithm corresponding to the following equation:

Y = Bmax * X/(KD + X);

where X is the concentration of the ligand, Y is the specific binding, and Bmax is the maximum binding expressed in the same units as the *y*-axis.

### 2.8. Aptamer-Mediated Pull-Down Assay

A total of 10 × 10^6^ MEC-1 cells were pre-treated with 0.4 µg/µL tRNA for 30 min at 37 °C in serum-free medium and then incubated with 400 nM of B85.T2 biotinylated aptamer or with a biotinylated unrelated aptamer used as a control (Ctrl Apt). Following three PBS washings, cells were lysed with 10 mmol/L Tris-HCl (pH 7.5) containing 200 mmol/L NaCl, 5 mmol/L EDTA, 0.1% Triton X-100, and protease inhibitors.

A total of 800 µg of cell lysates in 800 µL of lysis buffer were incubated with 200 µL of magnetic streptavidin beads (Promega Corporation, Madison, WI, USA) for 2 h with slow shaking. Magnetic beads were washed three times with lysis buffer, and bound proteins were recovered in Laemmli buffer and immunoblotted with anti-CD19 antibody (Cell Signaling Technology, Danvers, MA, USA).

### 2.9. Internalisation Assays

Internalisation assay by trypsin-EDTA washings and FACS analysis: A total of 5 × 10^5^ MEC-1 cells were washed three times with serum-free medium and then resuspended with 500 µL of serum-free medium containing 250 nM of FAM-labeled B85.T2 aptamer. Incubation with the aptamer was performed at different times (15 min, 1 h, and 2 h). Following incubation, cells were washed three times with ice-cold PBS to recover total aptamers or were incubated with 0.25% trypsin-EDTA for 30 min at 4 °C and then washed two times with ice-cold PBS to remove surface-bound aptamers not internalised. The mean fluorescence was measured at FACS, the cell auto-florescence was subtracted, and the aptamer internalisation rate was calculated.

Internalisation assay by immunofluorescence: A total of 4 × 10^5^ MEC-1 or Jurkat cells were pretreated for 30 min at 37 °C with slow shaking with 0.4 µg/µL tRNA and 100 nM of Ctrl Apt (Biotin), as unspecific competitors, in 400 µL of serum-free RPMI, and then FAM-labeled B85.T2 (5 µM) was added to cells in the presence of the competitors and incubated for two hours at 37 °C with slow shaking. Following incubation, cells were washed three times with ice-cold PBS, fixed with 4% PFA, and transferred in a dish of a 24-well plate containing a cover glass. The plate was incubated for 10 min at 37 °C, centrifuged for 5 min at 2000 r.p.m., and washed once with PBS; then, the cover glasses were mounted with SlowFade Diamond Antifade Mountant with DAPI (Life Technologies, Carlsbad, CA, USA) to mark nuclei

### 2.10. Aptamer-miRNA Conjugate Production

For aptamer–miRNA conjugation, the miRNA passenger and guide strands, resuspended in a specific buffer (20 mM 2-[4-(2-hydroxyethyl) piperazin-1-yl] ethane sulfonic acid (HEPES; pH 7.5), 150 mM NaCl, 2 mM CaCl_2_) were annealed by incubation at 95 °C for 10 min, 55 °C for 10 min, and 37 °C for 20 min. Further, the B85.T2 aptamer was correctly refolded by incubation at 85 °C for 5 min, on ice for 5 min, at 37 °C for 10 min, and then incubated with the passenger-guide miRNA strands at 37 °C for 30 min (ratio 1:1) to allow for the annealing of the stick sequences.

### 2.11. miRNA Delivery Assays

A total of 1 × 10^6^ cells were seeded in 12-well plates and treated with 400 nM of aptamers or conjugates. Following 24 h treatment, the cell culture medium was replaced with fresh medium without aptamers or conjugates. Total cell RNA was recovered 48 h after the starting of the treatment with TRIzol Reagent (Life Technologies, Carlsbad, CA, USA). A total of 50 ng of total RNA was then reverse-transcribed with gene-specific stem-loop reverse transcription primers and the TaqMan microRNA reverse-transcription kit (Life Technologies, Carlsbad, CA, USA), according to the manufacturer’s protocol. Amplification, to evaluate miRNA expression level, was performed with TaqMan miRNA Assays (Life Technologies, Carlsbad, CA, USA) on a Bio-Rad CFX384 Real-Time PCR Detection System (Bio-Rad, Hercules, CA, USA). U6 RNA was used as a reference gene.

### 2.12. Cell Viability and Propidium Iodide (PI) Staning

MEC-1 cells were seeded in 96-well plates (5 × 10^3^ cells per well) or 6-well plates (2 × 10^5^ cells per well) with or without treatment with 400 nmol/L aptamers or complexes to analyse cell viability or perform PI staining, respectively. After 72 h, treatments were renewed and incubation was prolonged for up to 5 days. Cell viability was monitored by CellTiter 96 Proliferation Assay (Promega, Madison, WI, USA), according to the manufacturer’s instructions. For PI staining, cells were recovered, washed with PBS, and incubated with 40 µg/mL PI (Sigma) for 15 min at room temperature. PI-positive cells were analysed with BD Accuri™ C6 cytometer.

## 3. Results and Discussion

### 3.1. Selection of Anti-CD19 Internalising Aptamers

For the selection of RNA aptamers specifically binding to human CD19 glycoprotein, we adopted a variant of the conventional cell-SELEX procedure, enriching for aptamers undergoing rapid intracellular uptake (‘cell-internalising SELEX’) [17].

**Figure 1:**
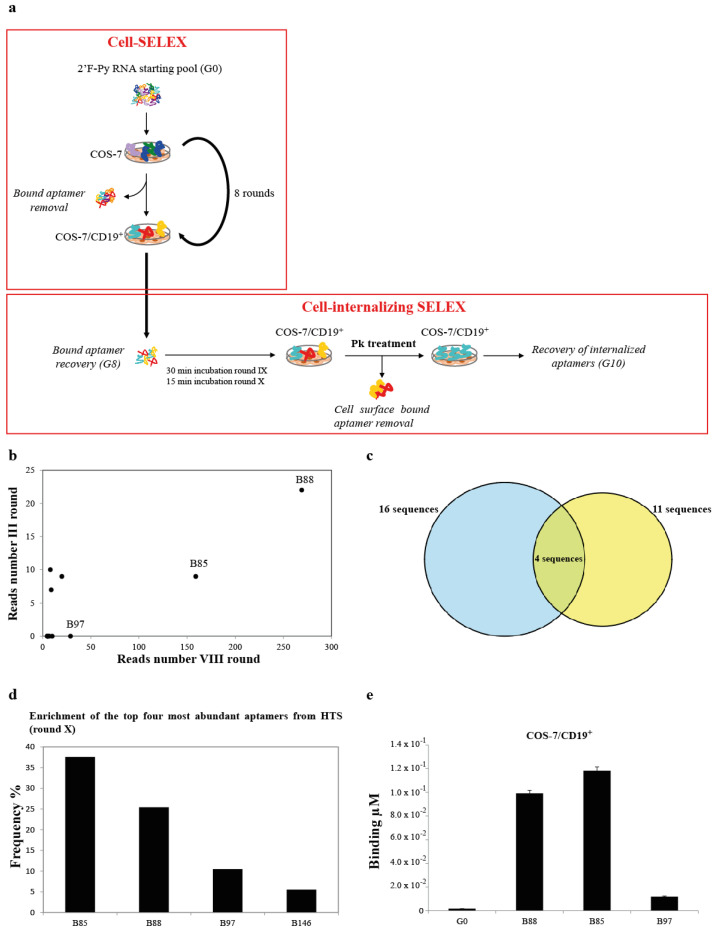
Cell-SELEX, HTS, and aptamer binding analysis. (**a**) Schematic representation of the cell-SELEX procedure used. Eight rounds of conventional cell-SELEX in which the aptamer pool was first incubated with parental COS-7 (CD19-) non-target cell (counter-selection step) and then with COS-7/CD19+ target cells (positive selection step) were performed. In the last two rounds (IX and X), a cell-internalising SELEX procedure in which, downstream, the positive selection step, a treatment with proteinase K (PK), was introduced to deplete for aptamers bound on the cell surface. Internalised aptamers were then recovered by RNA extraction and RT-PCR. (**b**) Scattering plot representing the aptamers with at least 5 reads resulting by Illumina deep sequence analyses at rounds III and VIII. (**c**) Venn diagram showing the overlap in aptamers with at least 5 reads in the HTS analysis between conventional cell-SELEX (round VIII, blue circle) and cell-internalising SELEX (round X, yellow circle). (**d**) Frequency of the top four most abundant aptamers from HTS at round X. (**e**) B88, B85, and B97 aptamers (200 nM) were incubated with COS-7/CD19^+^ cells for 30 min at 37 °C. G0 starting aptamer pool was used as a control. Binding values (μM) were measured by qRT-PCR. Error bars show the mean of technical duplicates ± SEM values.

As depicted in Figure 1a, for the first eight rounds, the positive selection steps were performed by incubating the RNA aptamer pool with COS-7 cells forced to express the full-length CD19 human receptor (COS-7/CD19^+^) (Figure S1). Each positive selection step was preceded by a counter-selection step, performed using parental COS-7 cells that do not express CD19 as a target. In order to enrich for cell-internalising aptamers, we then fulfilled two additional rounds (rounds IX and X) in which, downstream of the positive selection step, we introduced a treatment with proteinase K (PK), depleting for aptamers bound to CD19 expressed on the cell surface. Additional details regarding the conditions and the stringency applied at each SELEX round are reported in Table S1. To follow the enrichment throughout the selection procedure, we performed high-throughput sequencing (HTS) from round III to round X. The reads were filtered on the basis of the aptamer constant regions, and the read counts of the 100 most represented aptamers were analysed across all the SELEX rounds (Figure S2), pinpointing three aptamers, namely, B88, B85, and B97, as clearly more recurring. We considered the aptamers showing in round VIII in at least five reads each, which represented a group of 16 individual aptamer sequences. As shown, comparing the read number at round VIII with that at round III, we found that three of them, B88, B85, and B97 aptamers, were consistently enriched (Figure 1b). More precisely, these three aptamers represented about 82% of these most abundant individual sequences, with a frequency of 48.6% for B88, 28.7% for B85, and 5.2% for B97, indicating them as the most promising binders for human CD19 glycoprotein. Moreover, as shown in Figure S3a, in the phylogenetic dendrogram, three different families emerged, of which the B88, B85, and B97 aptamers were the most representative sequences. Further, the HTS analysis of the two additional rounds revealed that at round ×, four sequences (B85, B88, B97, and B146) survived to the internalisation rounds (Figure 1c and Figure S3b). These represent the top four most abundant aptamers at the last round, of which B85, B88, and B97 were the most ever represented sequences, with frequencies of about 37.5%, 25.4%, and 10.5%, respectively (Figure 1d). Thus, we tested these three aptamers for the effective binding to COS-7/CD19^+^. As evaluated by a reverse transcription quantitative polymerase chain reaction (RT-qPCR)-based binding assay, B88 and B85 were demonstrated to have an efficient binding activity, while no effective binding was detected for the B97 aptamer (Figure 1e).

These data indicate that, through the cell-SELEX protocol applied, we identified two 2′F-Py RNA aptamers, B88 and B85, able to effectively bind cells overexpressing the human CD19 glycoprotein.

### 3.2. B88 and B85 Aptamer Optimisation

**Figure 2:**
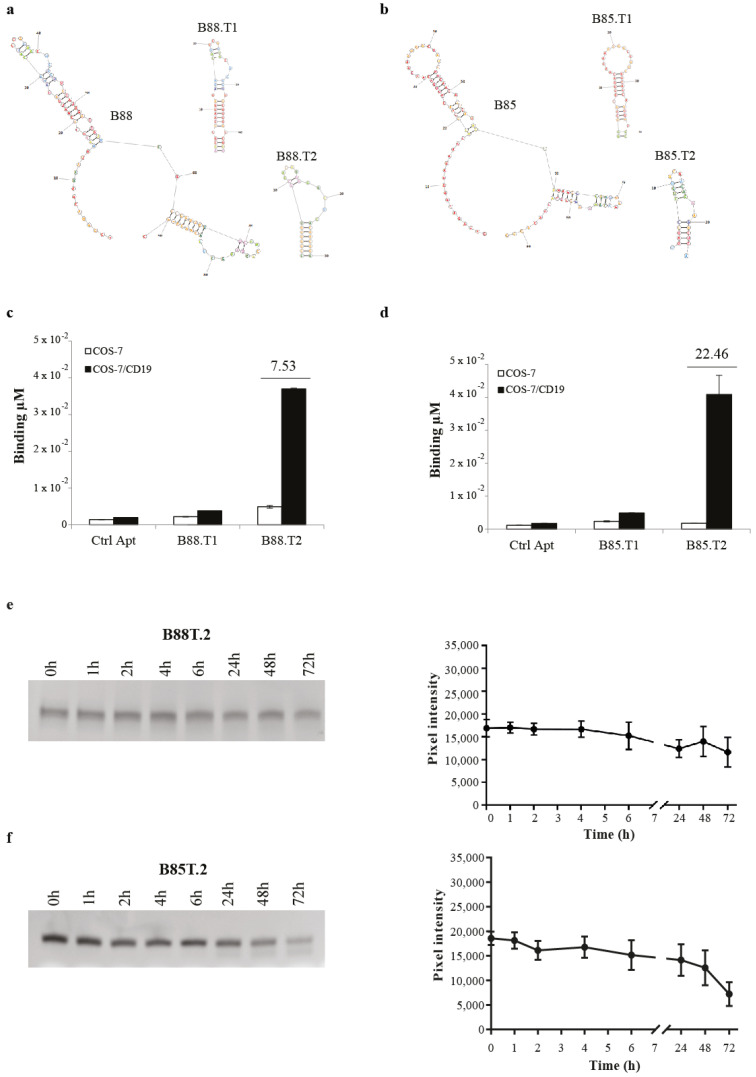
B88- and B85-derived short aptamers. (**a**,**b**) predicted secondary structures of B88 and B85 aptamers by RNA structure and design of two short versions for each aptamer (B88.T1, B88.T2, B85.T1, and B85.T2. (**c**,**d**) COS-7 and COS-7/CD19^+^ cells were treated with 200 nM of indicated aptamers for 30 min at 37 °C. Binding values (μM) were measured by qRT-PCR. Error bars show the mean of technical duplicates ± SEM values. (**e**,**f**) B88.T2 and B85.T2 serum stability. Aptamers (4 μM) were incubated in 80% human serum for indicated times. At each time point, RNA serum samples were collected and evaluated by electrophoresis on 15% denaturing polyacrylamide gel. Gels were stained with ethidium bromide and visualised at the Gel Doc EZ Imager. Gels representative of three independent experiments are shown (left panels). Original gels are available in Figure S8. Bands were quantified by using ImageJ program, and pixel intensity for each time point is reported in the graphs (right panels). Error bars show the mean of experimental triplicates ± SD values.

In order to optimise the selected aptamers for further development, we rationally designed shorter versions of both B88 and B85 on the basis of their secondary structures. To this end, we analysed the minimal free energy aptamer folding (by RNAstructure software available online at rna.urmc.rochester.edu/RNAstructure.html, accessed on 20 February 2021) and divided it into structured blocks that likely match with the aptamer binding sites. The ability of the corresponding truncated products to preserve the interaction with the human CD19 were then tested. On the basis of this strategy, we designed two structured truncated versions, B88.T1 and B88.T2, for the aptamer B88 (Figure 2a), and B85.T1 and B85.T2 for the aptamer B85 (Figure 2b). We then evaluated the truncated aptamers for their binding to COS-7/CD19^+^ cells and for the ability to efficiently discriminate them from parental COS-7 cells. To this purpose, we performed an RT-qPCR-based binding assay and observed that the B88.T2 (Figure 2c) and the B85.T2 (Figure 2d) short aptamers preserve the ability to bind COS-7/CD19^+^ target cells, effectively discriminating them from the parental CD19^−^ cell line, with a clearly higher discriminatory power for the B85.T2 aptamer, showing a fold increase of binding on COS-7/CD19^+^ over COS-7 of about 22, compared to about seven for the B88.T2 aptamer.

Resistance to nucleases present in human biological fluids is a major prerequisite for aptamer development. In our cell-SELEX protocol, we used a library of nuclease-resistant RNA aptamers modified with 2′F-Py. However, in order to test whether the selected molecules were adequately stable or needed additional chemical modifications, we evaluated their stability in human serum. To this purpose, the B88.T2 and B85.T2 aptamers were incubated at 37 °C in 80% human serum for increasing times, up to 72 h. Then, the integrity of RNA samples was analysed on denaturing polyacrylamide gel electrophoresis (Figure 2e,f, left panels), and the band intensities were quantified by using the ImageJ program and reported in the graphs (Figure 2e,f, right panels). As shown, both B88.T2 and 85.T2 aptamers remained almost completely stable at up to 6 h, and then started to gradually degrade, with predicted half-lives of approximately 100 and 60 h, respectively.

These data indicate that the B88.T2 and B85.T2 short aptamers hold an enhanced resistance to degradation in human serum. The B85.T2 aptamer, showing the best binding activity on COS-7 expressing human CD19 on the cell surface, was chosen for further characterisation analyses.

### 3.3. B85.T2 Aptamer In Vitro Binding Activity

**Figure 3:**
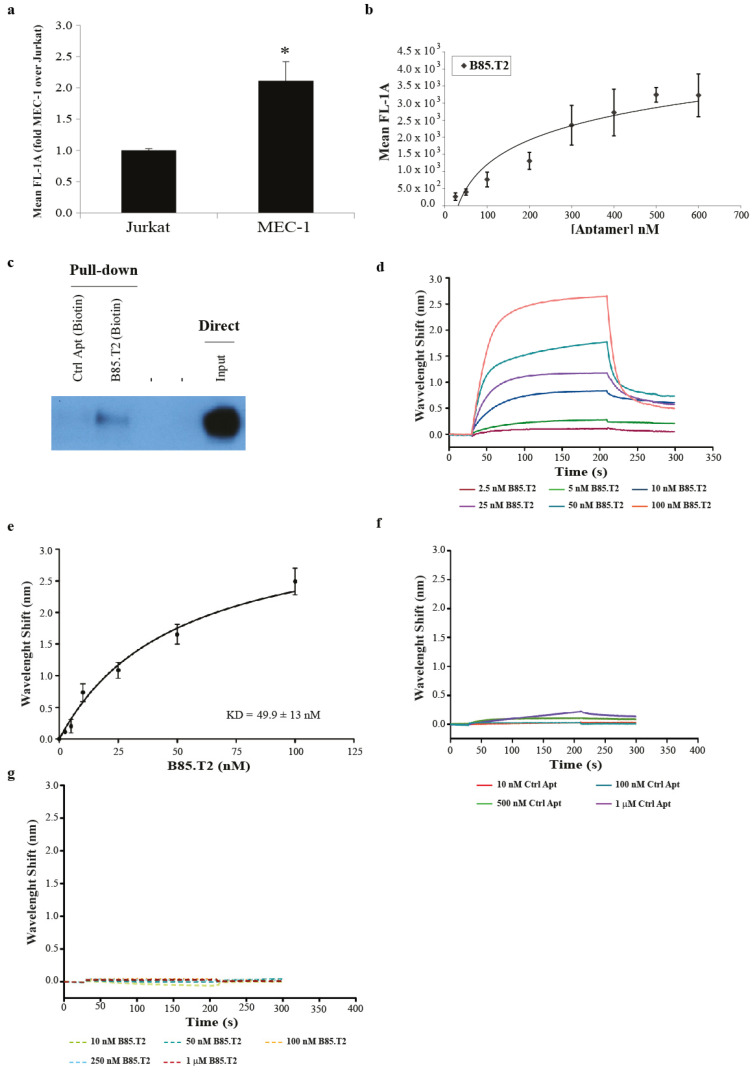
B85.T2 aptamer-binding activity. (**a**) Binding analysis of the B85.T2 aptamer on CD19^+^ MEC-1 cells and CD19^−^ Jurkat leukaemia cells. Cells were pre-treated for 30 min at 37 °C with slow shaking with 0.4 µg/µL tRNA and 100 nM of biotinylated Ctrl Apt as unspecific competitors. Then, the B85.T2 FAM-labelled aptamer (250 nM) was added to cells for 30 min at 37 °C with slow shaking in the presence of competitors. Mean fluorescence was measured by FACS, cell auto-florescence was subtracted, and data were reported in the graph as fold increase of aptamer binding on MEC-1 over Jurkat. Error bars show the mean of experimental triplicates ± SD values. Statistics was calculated using Student’s *t* test, * *p* < 0.05 (**b**) Dose-dependent binding of the B85.T2 aptamer on MEC-1 cells. Cells were incubated with increasing concentrations of FAM-labelled aptamer for 30 min at 37 °C in serum-free medium. The mean fluorescence was measured at FACS, cell auto-florescence was subtracted, and data were reported in the graph. Error bars show the mean of experimental triplicates ± SEM values. (**c**) Aptamer-mediated pull-down. MEC-1 cells were incubated with 400 nM of the biotinylated B85.T2 aptamer or with a biotinylated control aptamer. Cell lysates were purified on magnetic streptavidin beads (Promega Corporation, Wisconsin, USA) and immunoblotted with an anti-CD19 antibody. A total of 4 μg of total cell extracts from MEC-1 cells (Input) were loaded as a reference. The original Western Blot is available in Figure S8. (**d**) Dose–response BLI binding interferograms of B85.T2 at concentrations between 2.5 and 100 nM to purified rhCD19 immobilised on ARG2 sensor-chips. Data are representative of triplicate experiments. (**e**) Dose–response binding curve of BLI signals at plateau (202 s) as a function of aptamer concentration. Data represent the average of triplicate experiments ± standard deviation (SD). (**f**) Dose–response BLI binding interferograms of the control aptamer (Ctrl Apt) at concentrations between 10 nM and 1 µM to purified rhCD19 immobilised on ARG2 sensor chips. Data are representative of triplicate experiments. (**g**) Dose–response BLI binding interferograms of B85.T2 at concentrations between 10 nM and 1 µM to purified rhBCMA immobilised on ARG2 sensor chips. Data are representative of triplicate experiments.

In order to further characterise the B85.T2 aptamer, we evaluated its specific binding on leukaemia cells. To this purpose, we used a 5′ FAM-labelled version of the aptamer and analysed its binding activity on human CD19^+^ chronic B leukaemia cells (MEC-1) by FACS (fluorescence-activated cell sorting) using the immortalised T lymphocytes (Jurkat) from acute leukaemia, not expressing CD19, as a negative control cell line. The MEC-1 line arose from Epstein–Barr virus-carrying cells before the aggressive clinical stage within the subclone that was in the early prolymphocytic transformation [18]. Results show that the aptamer B85.T2 is able to discriminate MEC-1 from Jurkat cells, with a fold increase of binding on MEC-1 (CD19^+^) over Jurkat (CD19^−^) of about 2 (Figure 3a). In addition, to assess the selective interaction of B85.T2 with CD19, we also checked its binding on other control (CD19^−^) cancer cell models (Figure S4a), expressing different surface protein patterns. Importantly, the aptamer was able to significantly discriminate MEC-1 (CD19^+^) cells from glioblastoma (T98G, U87MG) and NSCLC (A549) cells (Figure S4b). Therefore, the aptamer maintained its discrimination ability on human malignant CD19^+^ B cells, although with a lower degree if compared to COS-7 cells forced to express the human CD19 glycoprotein (Figure 2d and Figure S5). This may have been due to various reasons, one of which is that COS-7 is the cell system used in the counter-selection step of the cell-SELEX. Therefore, the aptamer may exhibit a lower non-specific background of binding on this cell line compared to other cells.

Binding specificity on MEC-1 cells was further evaluated through performing a dose–response FACS analyses using FAM-labelled B85.T2 at concentrations ranging between 25 and 600 nM. Binding was dose-dependent and saturable for concentrations above 500 nM, thus suggesting it was not due to non-specific interactions (Figure 3b).

In addition, in order to demonstrate the direct interaction between the B85.T2 aptamer and the human CD19 receptor expressed on the B-chronic lymphocytic leukaemia (B-CLL) cell surface, we performed an aptamer-mediated affinity pull-down assay on streptavidin-coated beads by using the biotinylated B85.T2 aptamer. Briefly, MEC-1 (CD19^+^) B-CLL cells were treated with the biotinylated B85.T2 aptamer, and cell extracts were purified on streptavidin-coated beads, followed by immunoblotting with an anti-CD19 antibody. As shown in Figure 3c, the B85.T2 aptamer is able to interact with CD19 glycoprotein, whereas no binding was observed with an unrelated 2′F-Py RNA aptamer (Ctrl Apt) used as a negative control.

As a next step, the affinity of the B85.T2 aptamer with the purified human recombinant CD19 (rhCD19) glycoprotein was evaluated using BLI. Dose-dependent binding of B85.T2 aptamer to the immobilised purified rhCD19 at concentrations ranging between 2.5 and 100 nM were detected. By data fitting, a dissociation constant (K_D_) of 49.9 ± 13 nM (R^2^ = 0.9844) was estimated (Figure 3d,e), while no significant binding was detected with the unrelated aptamer (Ctrl Apt) (Figure 3f). Further, no significant binding was recorded between B85.T2 aptamer, tested up to 1 µM, and the unrelated rhBCMA purified protein (Figure 3g).

Collectively, our data indicate that the B85.T2 aptamer is a high-affinity and specific ligand for both the human native CD19 glycoprotein expressed on the surface of malignant B cells and its recombinant purified form.

### 3.4. B85.T2 Aptamer as a Carrier for miRNA Delivery

**Figure 4:**
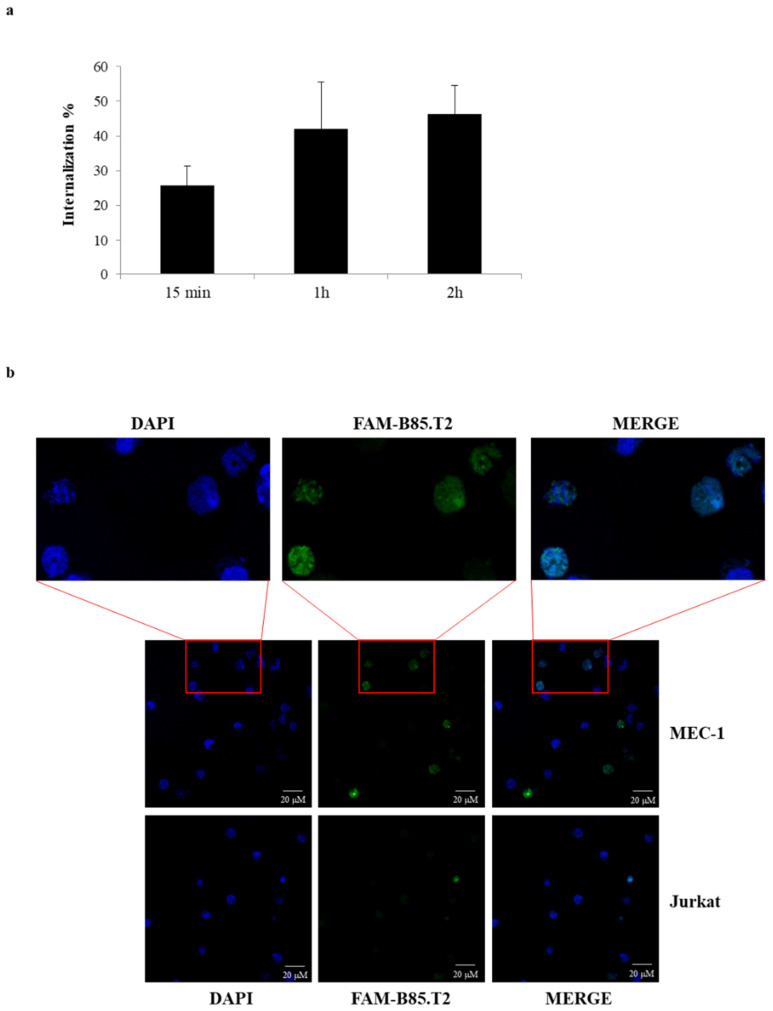
B85.T2 cell internalisation. (**a**) Internalisation of the B85.T2 aptamer on MEC-1 cells. FAM-labelled B85.T2 aptamer (250 nM) was incubated for the indicated times with cells, and then cells were recovered following three washings with PBS in order to recover total RNA, or one washing with trypsin-EDTA at 4 °C followed by two washings with PBS in order to remove cell surface bound aptamers not internalised. The quantities of the total and internalised aptamer were measured at FACS by evaluating the mean fluorescence of each point. The mean fluorescence was measured at FACS, the cell auto-florescence from untreated cells was subtracted, and the aptamer internalisation rates were calculated and reported in the graph as percentage (%) of aptamer internalisation. Error bars show the mean of experimental duplicates ± SD values. (**b**) Indicated cells were incubated at 37 °C for 2 h with 5 μM FAM-labelled B85.T2. After treatment, cells were fixed with 4% PFA, mounted with SlowFade Diamond Antifade Mountant with DAPI to mark nuclei, and then the images were taken with Zeiss LMS700 confocal microscopy (oil objective 63×, zoom 0.6). For MEC-1 cells, a higher magnification of each slice is also shown above. Images were processed equally to reduce the unspecific background. Scale bars depict 20 μm length.

As a next step, we investigated the capacity of the B85.T2 aptamer to efficiently internalise into MEC-1 CD19^+^ cells. To this purpose, we performed a FACS-based internalising assay by using the FAM-labelled B85.T2 aptamer. Cells were first incubated with the fluorescent aptamer for different times (15 min, 1 h, and 2 h) or left untreated. Then, cells were washed three times with ice-cold PBS to remove unbound or not specifically bound aptamers and recover total specific bound aptamers, or were incubated with 0.25% trypsin-EDTA for 30 min at 4 °C and then washed two times with ice-cold PBS to remove surface bound aptamers not internalised. The mean fluorescence was measured at FACS, the auto-florescence from untreated cells was subtracted, and the aptamer internalisation rates were calculated. As shown in Figure 4a, the B85.T2 aptamer rapidly internalises into MEC-1 CD19^+^ cells, reaching ≈ 50% internalisation following 2 h of treatment.

Aptamer binding and internalisation into human CD19^+^ B cells were further confirmed by immunofluorescence using confocal microscopy. As shown in Figure 4b, upon 2 h of incubation, the FAM-labelled B85.T2 aptamer was found into MEC-1 CD19^+^ cells but not into CD19^−^ Jurkat cells, in which only a slight background can be detected. The magnification of MEC-1 cells clearly indicates that the aptamer internalises into the cells reaching the nucleus, as demonstrated by the co-localisation with the DAPI. This is further confirmed by the Z-stack analysis (Figure S6).

**Figure 5:**
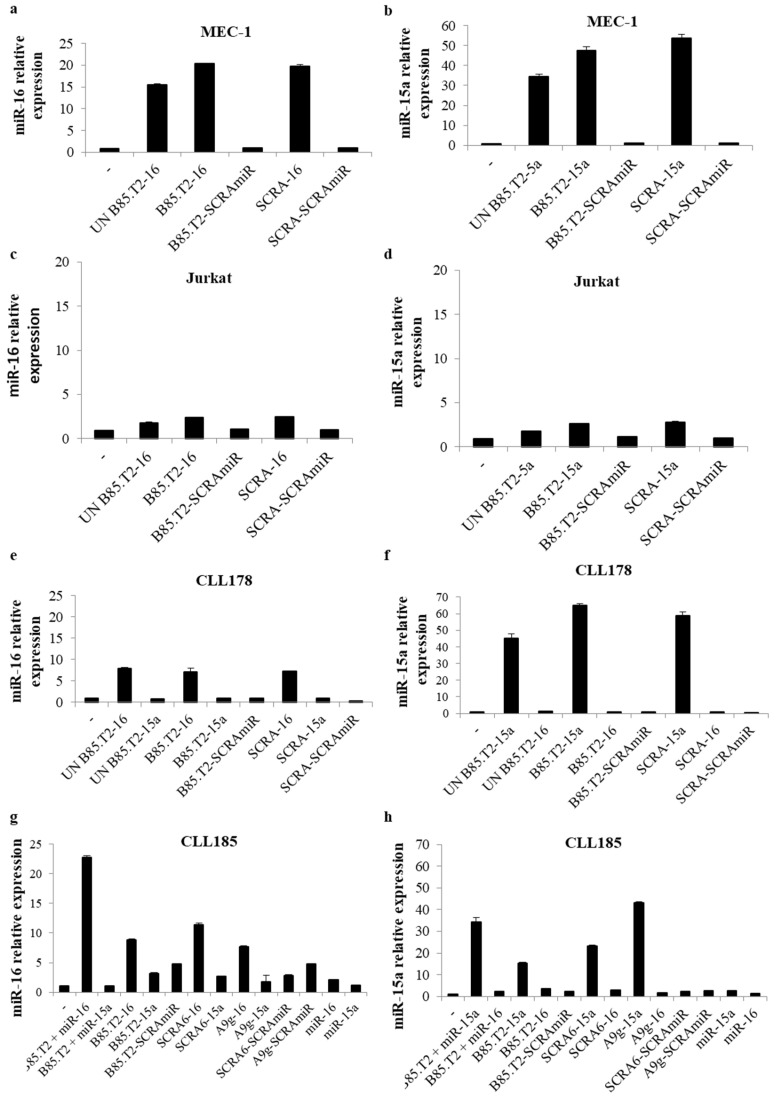
Aptamer miRNA delivery into CD19^+^ continuous and primary leukemic cells. (**a**,**b**) MEC-1 cells were treated with indicated molecules (400 nM) for 24 h. Then, cell culture medium was replaced with fresh medium without aptamers or conjugates and grown for an additional 24 h. MiR-16 (**a**) and miR-15a (**b**) levels were evaluated by qRT-PCR. U6 RNA was used as a reference gene. miRNA expression relative to cells treated with a control conjugate (SCRA-SCRAmiR) is reported in the graphs. Error bars show the mean ± SD values on technical replicates. (**c**,**d**) Jurkat cells were treated with indicated molecules (400 nM) for 24 h. Then, cell culture medium was replaced with fresh medium without aptamers or conjugates and grown for additional 24 h. miR-16 (**c**) and miR-15a (**d**) levels were evaluated by qRT-PCR. U6 RNA was used as a reference gene. miRNA expression relative to untreated cells is reported in the graphs. Error bars show the mean ± SD values on technical replicates. — (untreated cells); UN B85.T2-16 (unconjugated chimera B85.T2-miR-16); B85.T2-16 (chimera B85.T2-miR-16); B85.T2-SCRAmiR (control chimera with B85.T2 aptamer conjugated to a scrambled miR); SCRA-16 (control chimera with a control aptamer stick conjugated to miR-16); SCRA-SCRAmiR (control chimera with a control aptamer stick conjugated to a scrambled miR); UN B85.T2-15a (unconjugated chimera B85.T2-miR-15a); B85.T2-15a (chimera B85.T2-miR-15a); SCRA-15a (control chimera with a control aptamer stick conjugated to miR-15a). (**e**,**f**) CD19^+^ CLL patient-derived cells (CLL178) were treated with indicated molecules (400 nM) for 24 h. Then, cell culture medium was replaced with fresh medium without aptamers or conjugates and grown for an additional 24 h. miR-16 (**e**) and miR-15a (**f**) levels were evaluated by qRT-PCR. U6 RNA was used as a reference gene. miRNA expression relative to untreated cells is reported in the graphs. Error bars show the mean ± SD values on technical replicates. (**g**,**h**) CD19^+^ CLL patient-derived cells (CLL185) were treated with indicated molecules (400 nM) for 24 h. Then, cell culture medium was replaced with fresh medium without aptamers or conjugates and grown for an additional 24 h. miR-16 (**g**) and miR-15a (**h**) levels were evaluated by qRT-PCR. U6 RNA was used as a reference gene. miRNA expression relative to untreated cells is reported in the graphs. Error bars show the mean ± SD values on technical replicates. — (untreated cells); UN B85.T2-16 (unconjugated chimera B85.T2-miR-16); B85.T2-16 (chimera B85.T2-miR-16); B85.T2-SCRAmiR (control chimera with B85.T2 aptamer conjugated to a scrambled miR); SCRA-16 (control chimera with a control aptamer stick conjugated to miR-16); SCRA-SCRAmiR (control chimera with a control aptamer stick conjugated to a scrambled miR); SCRA6-16 (control chimera with a control aptamer stick conjugated to miR-16); SCRA6-15a (control chimera with a control aptamer stick conjugated to miR-15a); SCRA6-SCRAmiR (control chimera with a control aptamer stick conjugated to a scrambled miR); A9g-16 (control chimera with an unrelated A9g aptamer stick conjugated to miR-16); A9g-15a (control chimera with an unrelated A9g aptamer stick conjugated to miR-15a); A9g-SCRAmiR (control chimera with an unrelated A9g aptamer stick conjugated to a scrambled miR); B85.T2 + miR-16 (non-stick B85.T2 mixed with miR-16 stick); B85.T2 + miR-15a (non-stick B85.T2 mixed with miR-15a stick); miR-16 (miR-16 guide); miR-15a (miR-15a guide).

Then, we investigated the potential use of the B85.T2 aptamer as a carrier of secondary reagents. To this scope, by using a ‘stick-end-based approach’, previously described for the targeted delivery of RNA therapeutics [14,19], we designed two different conjugates. The 3′-end of the aptamer was conjugated with the passenger strand of either the mature human miR-16 or miR-15a (Figure S7), both reported to be tumour suppressors in B-cell chronic lymphocytic leukaemia [20,21]. Obtained results demonstrated that when CD19^+^ MEC-1 cells were treated with B85.T2-16 or B85.T2-15a conjugate (Figure 5a,b), an effective upregulation of the correspondent microRNAs was detected. However, we observed an unspecific cell uptake when cells were treated with the control conjugates, SCRA-16 and SCRA-15a, in which a scrambled aptamer sequence was conjugated to either miR-16 or miR-15a. The same result was observed when cells were treated with unconjugated B85.T2-16 (UN B85.T2-16) or with unconjugated B85.T2-15a (UN B85.T2-15a), in which the aptamer and the miRNA moieties were not annealed to each other but only mixed together, suggesting that miR-16 and the miR-15a duplexes were able to internalise into MEC-1 cells in an aptamer-independent manner. Upon treatment of Jurkat cells (Figure 5c,d), the B85.T2 aptamer was not able to efficiently deliver miR-16 and miR-15a, and no unspecific cell uptake was detected treating cells with SCRA-16, SCRA-15a, UN B85.T2-16, or UN B85.T2-15a control molecules. Further, the capacity of the aptamer to allow the delivery of miR-16 and miR-15a was investigated on two primary CD19^+^ CLL patient samples (CLL178 and CLL185). As shown in Figure 5e,f, when CLL178 cells were treated with B85.T2-16 or B85.T2-15a conjugates, we observed an effective upregulation of the correspondent miRNAs. Moreover, in this case, an unspecific cell uptake was detected, as demonstrated by the increased levels of the miRNAs when cells were treated with SCRA-16, SCRA-15a, UN B85.T2-16, or UN B85.T2-15a control molecules. Similar results were also observed on CLL185 cells (Figure 5g,h).

**Figure 6:**
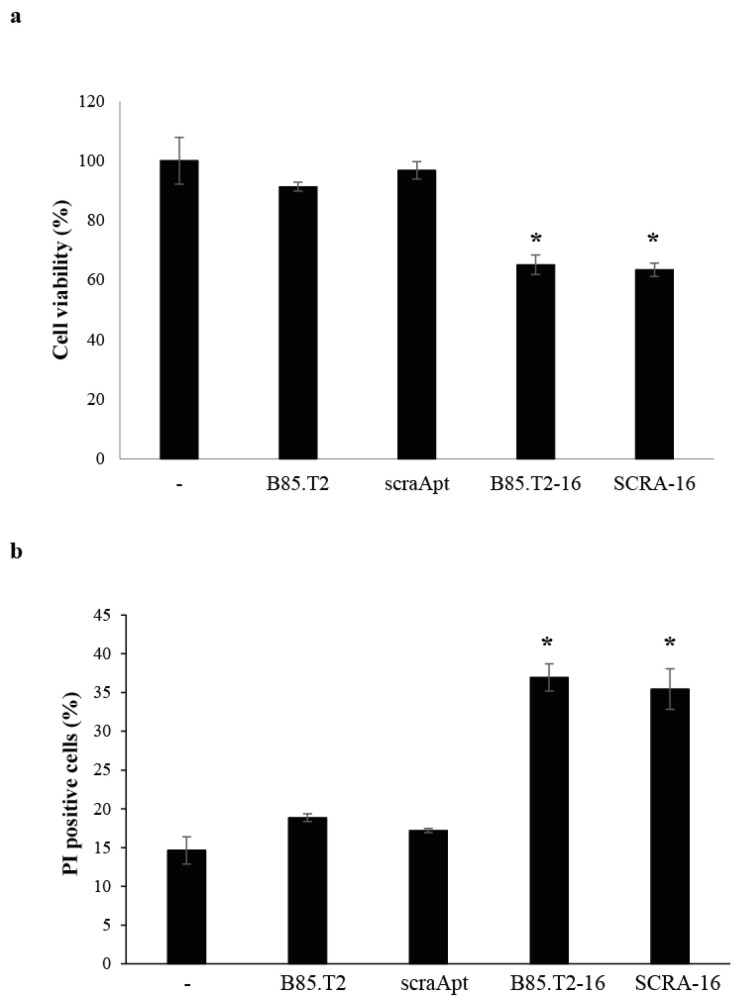
B85.T2-16 chimera functional activity. (**a**) MEC-1 cells were treated with indicated molecules (400 nM) for 5 days. Cell viability was monitored by MTT assay, and results are expressed as percent compared to untreated cells (-). Error bars show the mean ± SD values on technical triplicates. (**b**) MEC-1 cells were treated with indicated molecules (400 nM) for 5 days and then recovered and incubated with 40 µg/mL PI. The percentage of PI-positive cells was analysed at FACS and reported in the graph. Error bars show the mean ± SD values on experimental duplicates. Statistics were calculated using Student’s *t* test, * *p* < 0.05.

Furthermore, in order to support the therapeutic ability of B85.T2 conjugates, we analysed the functionality of B85.T2-16 on CD19^+^ MEC-1 cells. As shown in Figure 6, the treatment with the conjugate resulted in a significant reduction of cell viability (Figure 6a) and enhanced the cell death, as assessed by the increase of PI-positive cells (Figure 6b) after 5 days. In accordance with the miRNA levels, a similar effect was detected upon treatment with the SCRA-16 conjugate.

In a recent report, a CD19–RNA–aptamer complex was computationally constructed by molecular dynamics simulations, but no experimental investigation was performed on the identified sequences [22]. In addition, a DNA aptamer has been selected against the extracellular domain of the recombinant human CD19. This aptamer does not show sequence similarity with our aptamer and displays an apparent Kd value on purified protein around twofold lower [23].

Here, we selected a nuclease-resistant RNA aptamer, B85.T2, able to bind with high affinity and specificity to human CD19 glycoprotein expressed on COS-7 cell surface (Figure 2c,d) and to the recombinant human CD19 extracellular domain (Figure 3d,e). The B85.T2 aptamer demonstrated effectively discriminating human CD19^+^ chronic B leukaemia MEC-1 cells from CD19^−^ immortalised T lymphocyte Jurkat cells (Figure 3a), and to directly interact with CD19 glycoprotein expressed on the cell membrane with a high affinity (Figure 3b,c). Moreover, the aptamer showed an exquisite serum stability in vitro, adequate for further developments, without any additional chemical modifications (Figure 2f). Thus, this aptamer shows interesting features for the selective targeting of B-cell malignancies. In addition, it rapidly internalises into CD19^+^ target cells, reaching ≈ 50% of internalisation following 2 h treatment (Figure 4a). Although our data (Figure 4, Figure 5 and Figure 6) suggest the potential of the B85.T2 aptamer as a carrier for secondary RNA therapeutics into CD19^+^ B cells, this approach could be limited because of an unspecific cell uptake of structured RNAs. Given the high recognition specificity and affinity for CD19 of the aptamer we isolated, our findings provide a proof-of-concept for the future development of novel aptamer-based therapeutic compounds or bispecific immunotherapeutic strategies for B-cell malignancies.

## 4. Conclusions

Here, we selected a new 2′F-Py RNA aptamer, named B85.T2, binding with high affinity and specificity to both human CD19 glycoprotein expressed on cell surface and recombinant extracellular domain. Of note, for aptamer selection, we adopted a variant of the conventional cell-SELEX (‘cell-internalising SELEX’), which allowed us to isolate an aptamer able to effectively internalise into cells overexpressing the CD19 antigen on their surface. The selected aptamer shows an exquisite stability in human serum and was revealed to be a potential useful tool for CD19 targeting and for the delivery of secondary reagents in CD19^+^ cells.

The manuscript provides a proof-of-concept study that paves the way to the rational design of novel aptamer-based therapeutic compounds or bispecific immunotherapeutic strategies for B-cell malignancy treatment.

## Data Availability

Data are contained within the article or in the Supplementary Materials.

## Supplementary Materials

The following are available online at https://www.mdpi.com/article/10.3390/cancers13205220/s1, Figure S1: Human CD19 glycoprotein induced expression in COS-7 cells used in the positive selection step of the cell-SELEX procedure, Figure S2: Amplification recurrence, Figure S3: Phylogenetic dendrograms, Figure S4: B85.T2 aptamer ability to discriminate MEC-1 (CD19+) chronic B leukaemia cells from other cancer cell models (CD19−), Figure S5: CD19 expression in different cell lines, Figure S6: B85.T2 aptamer internalisation into MEC-1 CD19^+^ cells by immunofluorescence, Figure S7: Schematic representation of the generated stick-end-based aptamer constructs, Figure S8: Original Gels and Western Blots, Table S1: Cell-SELEX conditions.
