# Supplementary material for: Selection of a Nuclease-Resistant RNA Aptamer Targeting CD19
Source: Cancers (Basel). 2021 Oct 18;13(20):5220. doi: 10.3390/cancers13205220 (PMC8533794; doi:10.3390/cancers13205220)
Supplement: Supplementary file 1 [file cancers-13-05220-s001.zip › cancers-1359130-supplementary.pdf]

Supplementary Material: Selection of a Nuclease-Resistant RNA Aptamer Targeting CD19

Carla L. Esposito, Katrien Van Roosbroeck, Gianluca Santamaria, Deborah Rotoli, Annamaria Sandomenico, William G. Wierda, Alessandra Ferrajoli, Menotti Ruvo, George A. Calin, Vittorio de Franciscis and Silvia Catuogno

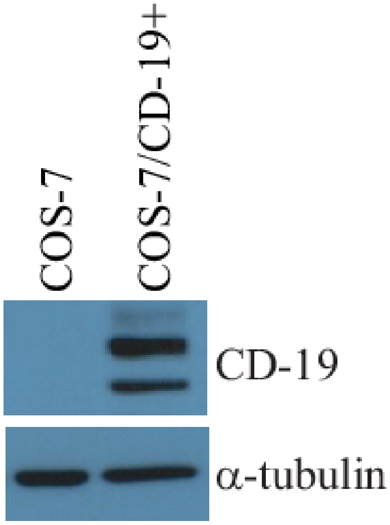

Figure S1. Human CD19 glycoprotein induced expression in COS-7 cells used in the positive selection step of the cell-SELEX procedure. 48 h post-transfection, 20 μg of lysates from parental COS-7 or transfected COS-7 (COS-7/CD19^{+}) were separated by electrophoresis on 10% SDS-PAGE and then blotted onto polyvinylidene difluoride membranes by electrophoretic transfer. Filter was immunoblotted with anti-CD19 antibody. Anti-β-actin antibody was used for normalization.

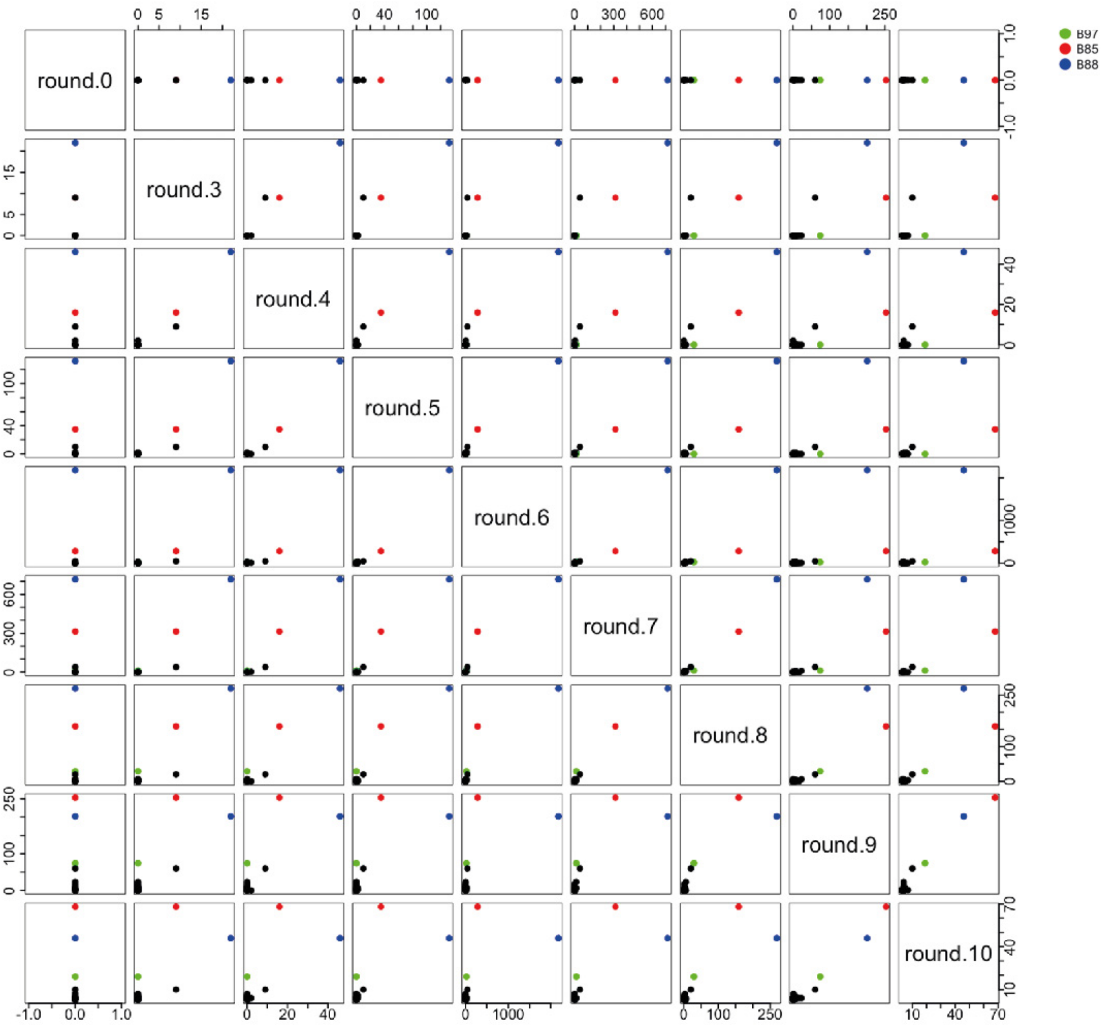

Figure S2. Amplification recurrence. Correlation plot of aptamers read count across all over SELEX rounds. Most enriched aptamers are color-coded.

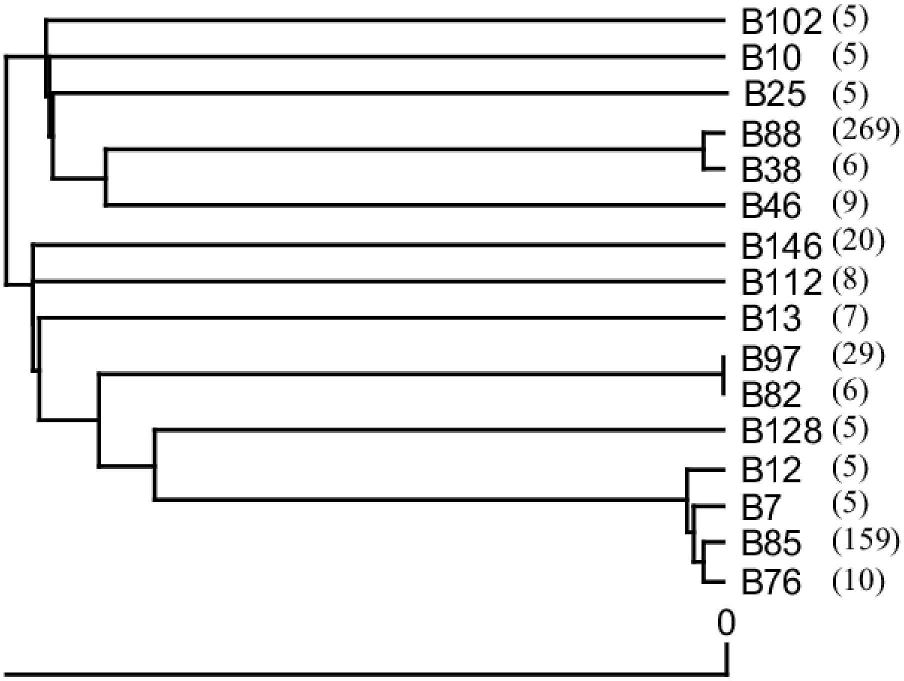

(a)

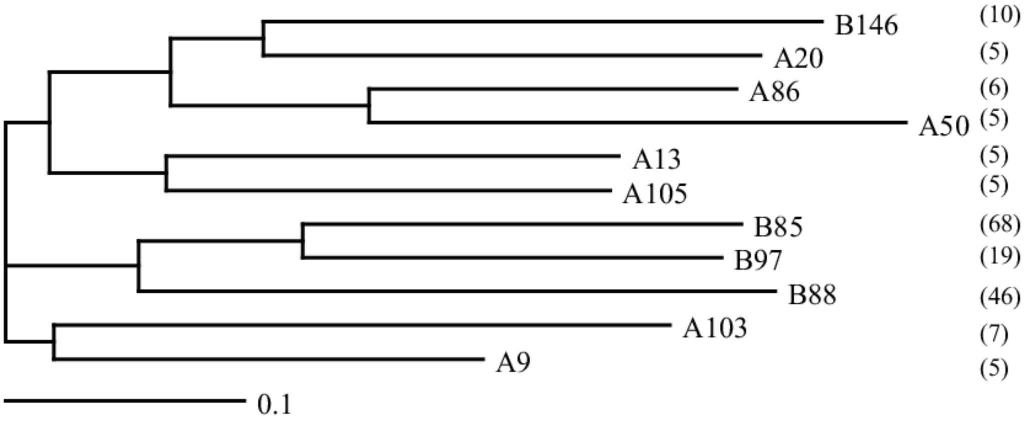

(b)

Figure S3. Phylogenetic dendrograms. Dendrograms by Clustal Omega Multiple Sequence Alignment tool by EMBL-EBI of the aptamer sequences with at least 5 reads in the HTS analysis at round VIII (**a**) and X (**b**). Number of reads for each aptamer is indicated in parentheses.

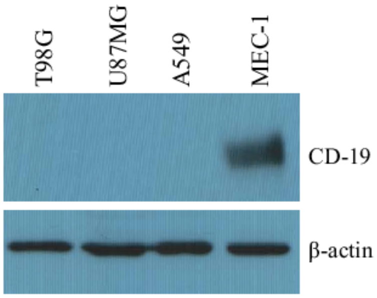

(**a**)

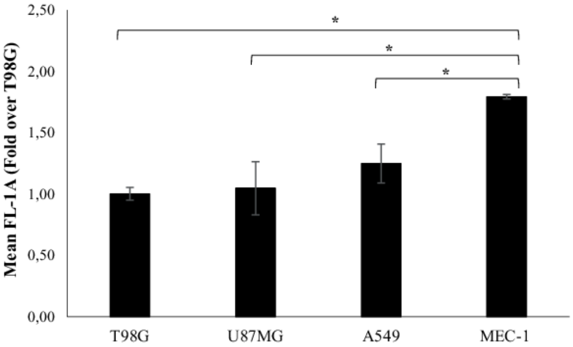

(**b**)

Figure S4. B85.T2 aptamer ability to discriminate MEC-1 ($CD19^+$) chronic B leukemia cells from other cancer cell models ($CD19^-$). (**a**) Expression levels of CD19 in different tumor models by Western Blot analysis. Cell lysates from T98G, U87MG (glioblastoma), A549 (NSCLC) and MEC-1 (chronic B cell leukemia) cells were loaded on 10% SDS-PAGE and separated by electrophoresis. Proteins were then blotted onto polyvinylidene difluoride membranes by electrophoretic transfer. Filter was immunoblotted with anti-CD19 antibody. Anti-β-actin antibody was used for normalization. (**b**) T98G, U87MG (glioblastoma), A549 (NSCLC) and MEC-1 (chronic B cell leukemia) cells were pretreated for 30 min at 37 °C with slow shaking with 0.4 µg/µl tRNA and 100 nM of biotinylated Ctrl Apt as unspecific competitors. Then the B85.T2 FAM-labeled aptamer (250 nM) was added to cells for 30 min at 37 °C with slow shaking, in the presence of competitors. Mean fluorescence was measured by FACS, cell auto-florescence was subtracted and data were reported in the graph as fold increase of aptamer binding on T98G cells. Error bars show the mean of experimental duplicates ± SD values. Statistics were calculated using Student's t test, *$p < 0.05$.

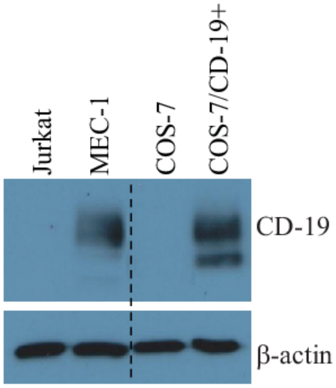

Figure S5. CD19 expression in different cell lines. 20 µg of lysates from Jurkat, MEC-1, parental COS-7 or transfected COS-7 (COS-7/$CD19^+$) cells 48 h post-transfection were separated by electrophoresis on 10% SDS-PAGE and then blotted onto polyvinylidene difluoride membranes by electrophoretic transfer. Filter was immunoblotted with anti-CD19 antibody. Anti-β-actin antibody was used for normalization.

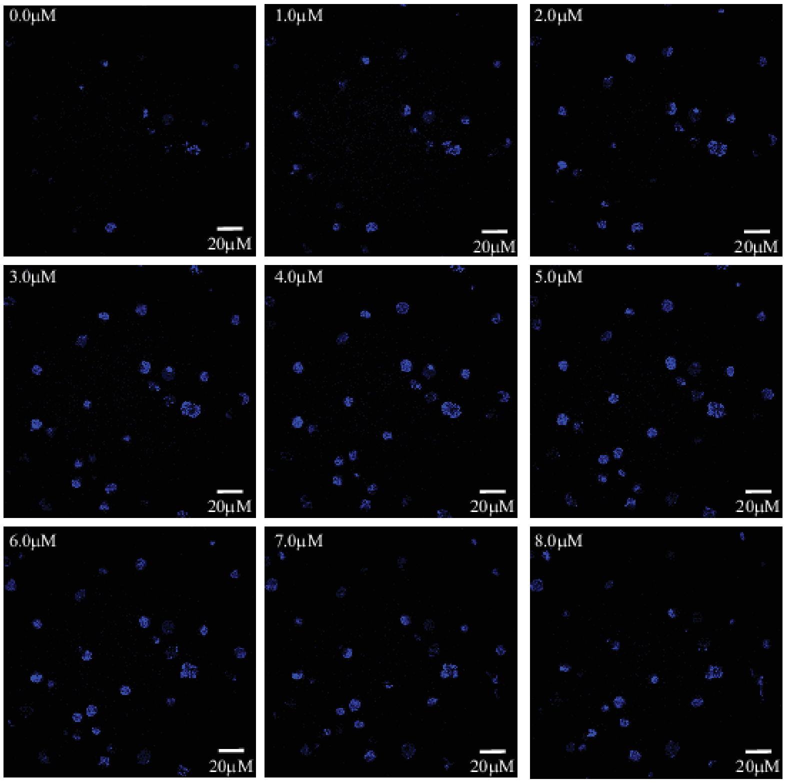
0.0μM
20μM
1.0μM
20μM
2.0μM
20μM
3.0μM
20μM
4.0μM
20μM
5.0μM
20μM
6.0μM
20μM
7.0μM
20μM
8.0μM
20μM

(**a**)

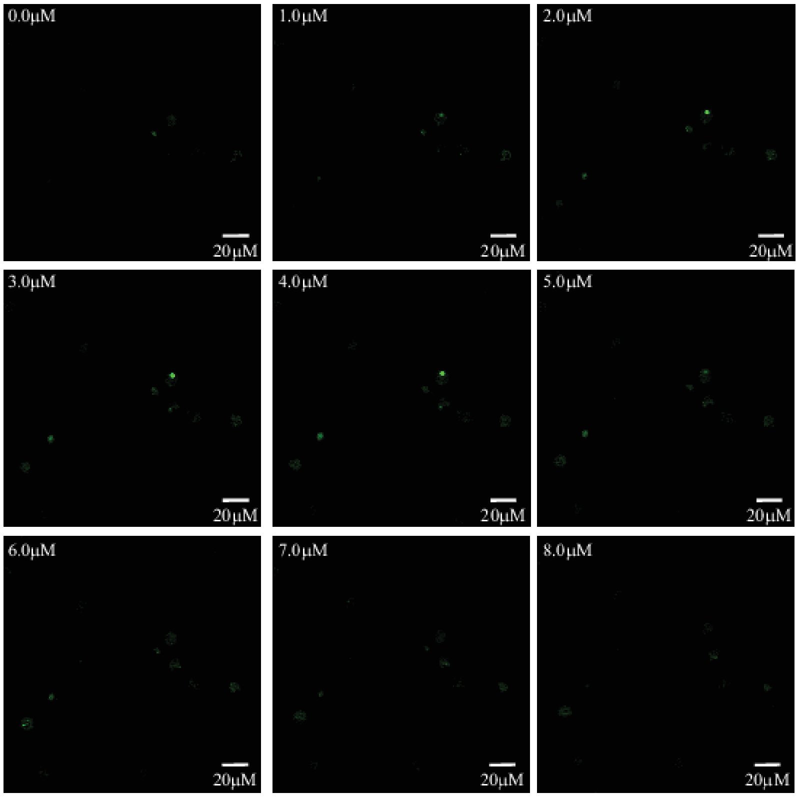
0.0μM
20μM
1.0μM
20μM
2.0μM
20μM
3.0μM
20μM
4.0μM
20μM
5.0μM
20μM
6.0μM
20μM
7.0μM
20μM
8.0μM
20μM

(**b**)

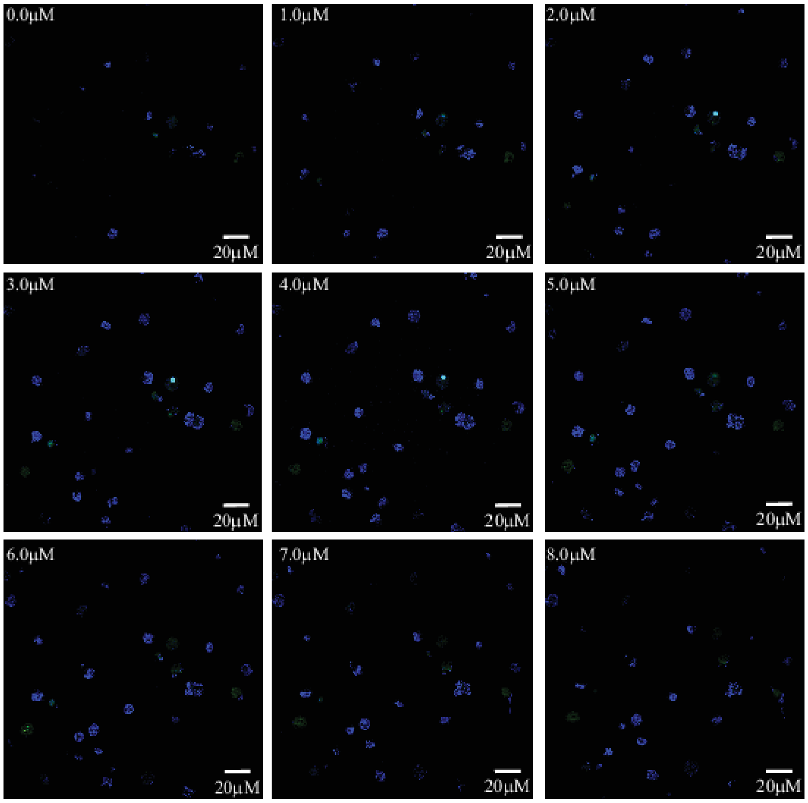

(**c**)

Figure S6. B85.T2 aptamer internalization into MEC-1 CD19+ cells by immunofluorescence. Cells were incubated at 37 °C for 2 hours with 5 µM FAM-labeled B85.T2. After treatment, cells were fixed with 4% PFA, mounted with SlowFade Diamond Antifade Mountant with DAPI to mark nuclei, and then the images were taken at Zeiss LMS700 confocal microscopy (oil objective 63x). 8-micron cell section was obtained using Z-stacking acquisition, where each slide represented has 1 µm thickness. Images have been processed equally to reduce unspecific background. DAPI staining (**a**), FAM (**b**) and merge (**c**) are shown. Scale bars depict 20 µm length. Punctate spots are typical of intracellular localization.

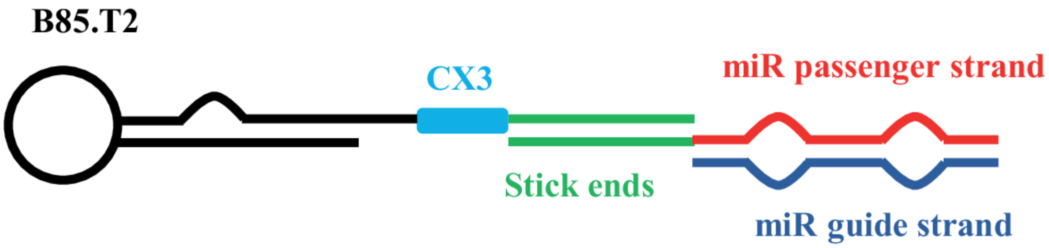

Figure S7. Schematic representation of the generated stick-end based aptamer constructs. The 3′ end of the B85.T2 aptamer was modified with the addition of a three-carbon linker spacer (CX3), and elongated with a chain containing 17 specific ribonucleotides. The 3′ end of the miRNA passenger strand was also elongated with 17 ribonucleotides showing a perfect complementarity with the chain added at the 3′ end of the aptamer, thus allowing sequence annealing.

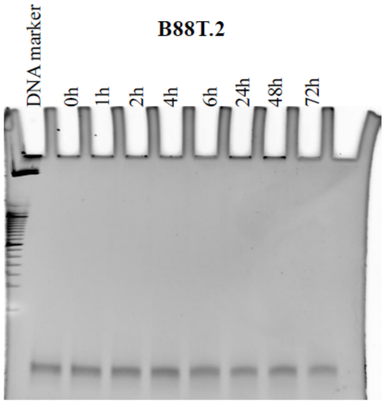

Figure 2E

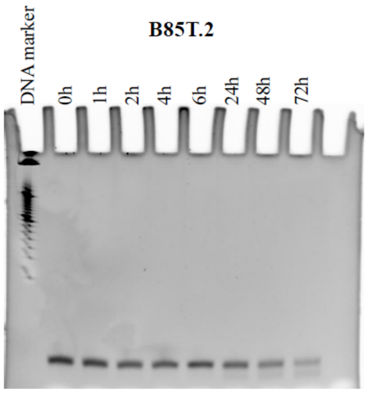

Figure 2F

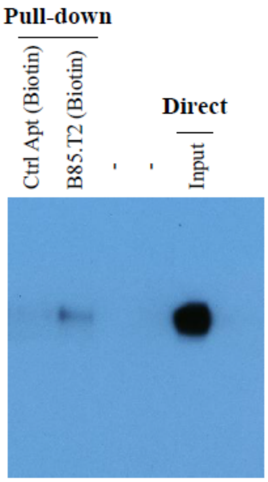

Figure 3c

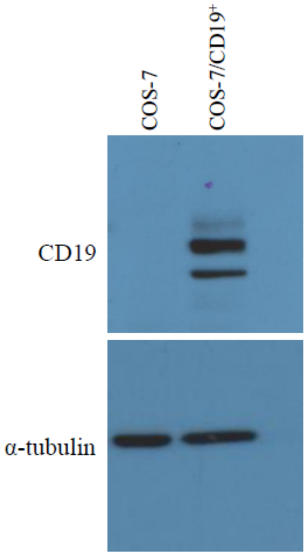

Figure S1b

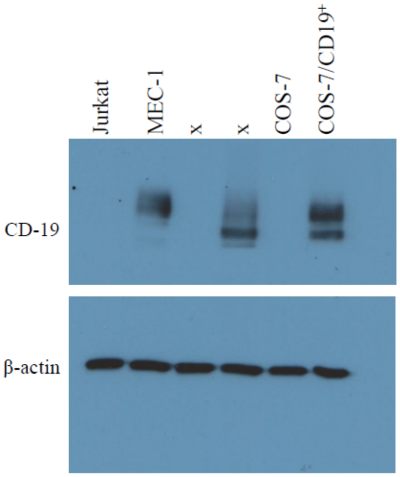

Figure S4

Figure S8. Original Gels and Western Blots.

Table S1. Cell-SELEX conditions.

Round	Aptamer pool amount (pmoles)	Number of cells at the day of incubation	Number of washes downstream the positive selection step	Number of counter-selection steps	Incubation time (min)	PK treatment
I	600	3.5×10^6	1	1	30 selection step<br>30 counter-selection step	No
II	600	3.5×10^6	2	1	30 selection step<br>30 counter-selection step	No
III	300	3.5×10^6	2	1	30 selection step<br>30 counter-selection step	No
IV	300	3.5×10^6	3	1	30 selection step<br>30 counter-selection step	No
V	300	3.5×10^6	4	1	30 selection step<br>30 counter-selection step	No
VI	300	1.8×10^6	4	1	30 selection step<br>30 counter-selection step	No
VII	300	1.8×10^6	5	1	15 selection step<br>30 counter-selection step	No
VIII	300	1.8×10^6	5	2	15 selection step<br>15 counter-selection step	No
IX	300	1.8×10^6	5	/	30	Yes
X	300	1.8×10^6	5	/	15	Yes
